# Can an mhealth clinical decision-making support system improve adherence to neonatal healthcare protocols in a low-resource setting?

**DOI:** 10.1186/s12887-020-02378-1

**Published:** 2020-11-27

**Authors:** Hannah Brown Amoakoh, Kerstin Klipstein-Grobusch, Irene Akua Agyepong, Mary Amoakoh-Coleman, Gbenga A. Kayode, J. B. Reitsma, Diederick E. Grobbee, Evelyn K. Ansah

**Affiliations:** 1 Julius Global Health, Julius Center for Health Sciences and Primary Care, University Medical Centre, Utrecht University, Utrecht, The Netherlands; 2 School of Public Health, University of Ghana, P.O. Box LG13, Legon Accra, Ghana; 3 Division of Epidemiology and Biostatistics, School of Public Health, Faculty of Health Sciences, University of the Witwatersrand, Johannesburg, South Africa; 4 Research and Development Division, Ghana Health Service, Dodowa Accra, Ghana; 5 Noguchi Memorial Institute, University of Ghana, Legon Accra, Ghana; 6 International Research Centre of Excellence, Institute of Human Virology, Abuja, Nigeria; 7 University of Health and Allied Sciences, Ho, Ghana

**Keywords:** Health care delivery, Neonatal health, Ghana, mHealth, Developing countries, Jaundice, Asphyxia, Sepsis

## Abstract

**Background:**

This study assessed health workers’ adherence to neonatal health protocols before and during the implementation of a mobile health (mHealth) clinical decision-making support system (mCDMSS) that sought to bridge access to neonatal health protocol gap in a low-resource setting.

**Methods:**

We performed a cross-sectional document review within two purposively selected clusters (one poorly-resourced and one well-resourced), from each arm of a cluster-randomized trial at two different time points: before and during the trial. The total trial consisted of 16 clusters randomized into 8 intervention and 8 control clusters to assess the impact of an mCDMSS on neonatal mortality in Ghana. We evaluated health workers’ adherence (expressed as percentages) to birth asphyxia, neonatal jaundice and cord sepsis protocols by reviewing medical records of neonatal in-patients using a checklist. Differences in adherence to neonatal health protocols within and between the study arms were assessed using Wilcoxon rank-sum and permutation tests for each morbidity type. In addition, we tracked concurrent neonatal health improvement activities in the clusters during the 18-month intervention period.

**Results:**

In the intervention arm, mean adherence was 35.2% (SD = 5.8%) and 43.6% (SD = 27.5%) for asphyxia; 25.0% (SD = 14.8%) and 39.3% (SD = 27.7%) for jaundice; 52.0% (SD = 11.0%) and 75.0% (SD = 21.2%) for cord sepsis protocols in the pre-intervention and intervention periods respectively. In the control arm, mean adherence was 52.9% (SD = 16.4%) and 74.5% (SD = 14.7%) for asphyxia; 45.1% (SD = 12.8%) and 64.6% (SD = 8.2%) for jaundice; 53.8% (SD = 16.0%) and 60.8% (SD = 11.7%) for cord sepsis protocols in the pre-intervention and intervention periods respectively. We observed nonsignificant improvement in protocol adherence in the intervention clusters but significant improvement in protocol adherence in the control clusters. There were 2 concurrent neonatal health improvement activities in the intervention clusters and over 12 in the control clusters during the intervention period.

**Conclusion:**

Whether mHealth interventions can improve adherence to neonatal health protocols in low-resource settings cannot be ascertained by this study. Neonatal health improvement activities are however likely to improve protocol adherence. Future mHealth evaluations of protocol adherence must account for other concurrent interventions in study contexts.

## Background

The Sustainable Development Goals aim to reduce the current high global neonatal mortality from 18 per 1000 to at least 12 per 1000 live births by 2030 [1]. Concerted effort is being harnessed through many international, national, district and community collaborations to make this a reality particularly in low-resource settings like sub-Saharan Africa and Southern Asia which contribute most to the global burden of neonatal mortality [2, 3]. Infections, birth asphyxia and prematurity contribute to the majority of neonatal deaths in low-resource settings (90%) [4]. Although morbidity and mortality from these conditions are largely preventable, the scarcity of health resources (facilities, personnel, basic equipment and medicines, training programmes, protocols etc.), allow these preventable deaths to thrive in the health systems of poorly resourced countries.

### Mobile health

(mhealth) is a potential tool to improve the efficiency of health workers and the health system as a whole in low-resource settings [5]. Many mHealth interventions have been applied in areas of vaccination, management of tuberculosis and HIV, monitoring of antenatal health services for pregnant women in low-resource settings and have been documented to have variable but largely good success [6–9]. In the field of clinical decision-making support (CDMS), few mHealth interventions have been implemented in low-resource settings [9–16], and even fewer studies report adherence to protocols or algorithms specified by these electronic CDMS systems.

Ghana is a lower middle-income country that reports high neonatal mortality rates of 25 per 1000 live births [17]. Non-adherence to standard clinical protocols has been identified as a cause of Ghana’s high neonatal mortality [18, 19]. Previous studies have shown the absence of standard health protocols (the Safe Motherhood Protocol) for about 44% of health workers at the point of service delivery [20]. To bridge this protocol access gap, the Ghana Health Service (GHS) in collaboration with her Dutch partners designed and implemented an mHealth clinical decision-making support system (mCDMSS) aiming to improve clinical decision-making and ultimately neonatal health outcomes that was tested in a cluster randomized controlled trial (CRCT) in the Eastern Region of Ghana [21].

### Description of the intervention

The mobile clinical decision making support intervention (THE INTERVENTION for short for the rest of this paper) consisted of 4 components - phone calls (voice), text messaging (SMS), access to the internet (data) and access to an unstructured supplementary service data (USSD) that provided emergency protocols in response to selection from a short code drop-down menu. The messages on the USSD were created by a design team of frontline health workers, family physicians, obstetricians and paediatricians in the Greater Accra Region, drawing on Ghana’s Safe Motherhood Protocols [22]. All four components of the intervention were part of a single composite intervention delivered on a non-smart mobile phone. Researchers considered access to the USSD the main intervention component. Health workers were expected to use the phones primarily to access neonatal and maternal health emergency protocols via the USSD and obtain additional support from colleagues and the internet via the other intervention components. Each project mobile phone had a unique Subscriber Identification Module (SIM) card. All the SIM cards were networked in a Closed User Group (CUG) that allowed free and unlimited access to the USSD. Access to the intervention was, however, limited to the project SIM cards to avoid contamination. Health workers were trained on how to use the intervention firstly at a group gathering in each intervention district capital before the start of the CRCT and then at least once during monitoring visits in their individual health facilities during intervention implementation. Monthly reminders were also sent to health workers about the availability of the USSD platform for their use.

### Study objectives

Our objectives in this study were to assess the quality of neonatal healthcare in the Eastern Region of Ghana, by examining the change in health worker adherence to neonatal health protocols in both study arms of the CRCT from a pre-intervention period to an intervention implementation period, and to investigate differences in adherence within and between the study arms during these time frames. We also assessed whether and which concurrent neonatal health improvement activities (not related to the intervention) occurred during the trial period. **This study will provide insights to explain the observed effect of the intervention on neonatal mortality**. **For brevity, we limited this study to the neonatal component of the CRCT. Adherence to maternal health protocols during the CRCT will be published separately.**

## Methods

### Study design & setting

We designed a longitudinal study and performed a cross-sectional document review within two clusters selected from each arm of a cluster randomized trial at two different time points: before and during the trial. The trial aimed to assess the impact of an mCDMSS on neonatal mortality in the third most populous region in Ghana- the Eastern Region [23]. **The trial was registered at** **clinicaltrials.gov****(trial identifier number** ***NCT02468310*) and the Pan African Clinical Trials Registry (trial identification number** ***PACTR20151200109073)*.**

The Eastern Region has a neonatal mortality rate of 30 per 1000 live births and ranks fourth highest in terms of neonatal mortality in Ghana [24]. The region was divided into twenty-one (21) geographic local administrative units called districts at the time of the study. The CRCT was implemented over 18-months (August 2015 to January 2017) in 16 of these districts randomized into 8 intervention and 8 control clusters. Each of the 16 districts formed one cluster of the CRCT. The CRCT has been previously described elsewhere [25].

### Sampling of clusters

For logistic reasons, one well-resourced and one poorly-resourced cluster were purposively selected from each CRCT arm making two clusters per arm. The selection criteria were based on the number and mix of health facilities (hospitals, community health planning and services compounds (CHPS), health centres (HCs) and maternity homes) in the district and the midwife to the number of deliveries (per annum) ratio in a district (reference year was 2014). Following cluster selection, district hospitals were sampled because initial assessment showed that almost all cases of neonatal morbidity of interest in this study were managed in the district hospitals.

### Recruitment of study participants

All cases of in-patient neonatal morbidities of birth asphyxia, jaundice and cord sepsis that were managed in the district hospitals 9 months before the intervention started and, 9 months to the end of the intervention implementation period were studied to assess health worker adherence to protocols regarding morbidities. These morbidities were selected as the most common causes of neonatal morbidity in the study setting [26].

### Data collection

Baseline data regarding the number and category of health workers providing neonatal health services in each cluster was collected using a checklist. We extracted data concerning the number of deliveries per study cluster during the two-time frames of interest from the district health information management system 2 (DHIMS2). The DHIMS2 is a data recording, collection, collation and analysis tool that hosts the entire national institutional health data of Ghana [21].

We utilized a scoring system based on existing health protocols as done in previous studies [27–29]. In each hospital, the head of the maternity or paediatric unit and the health information manager were contacted to identify the medical records (registers and books) that are routinely used in the hospitals to document in-patient neonatal data. A list of all in-patient cases of birth asphyxia, jaundice and cord sepsis was then populated from the ‘in-patient admissions and discharge register’ which documents all admitted cases in a hospital. A document review of the management of these cases was done using a checklist to assess health worker adherence to neonatal protocols. This checklist was based on Ghana’s Safe Motherhood Protocol for management of neonatal morbidities. During data extraction, protocol items were assessed under the following themes where applicable for each morbidity type: i. Diagnosis (e.g., ‘Diagnosis documented’), ii. Signs and symptoms of disease (e.g., ‘Colour of baby’, ‘Cord assessed for odour, pus and wetness’), iii. Investigation (e.g., ‘Serum bilirubin checked’) iv. Treatment given (e.g., ‘Airway of baby cleared through suction’, ‘Phototherapy given, or sunbath advised’, ‘Antibiotics given’). Assessment of adherence was only done for items that are considered mandatory in the management of each morbidity type (Tables 2, 3, 4). Additional file 1 details the type of facility records utilized in the data collection process.

Data concerning other concurrent neonatal health improvement interventions such as trainings and workshops that took place in the study districts during the 18-month intervention period was collected using a checklist. The district public health nurse in each cluster and the in-service trainers of the four hospitals assisted in extracting the relevant data from their facility record books.

### Data analysis

The data were checked for errors, cleaned and analyzed at health facility level. We calculated the number of deliveries per midwife and doctor to estimate the delivery related workload in the study clusters. Descriptive analysis of neonatal data was performed. Items for each morbidity protocol was scored as ‘adhered to’ and assigned a score of 1 if there was written documentation of adherence to the item in any of the medical records. A protocol item was scored ‘not adhered to’ and assigned a score of zero (0) only when there was no written documentation of adherence in all the medical records. When the records of a neonate could not be traced because a register or book for a neonate was not found, protocol items were scored as ‘don’t know’ and assigned a score of zero (0). Additional file 1 details the ‘don’t know’ responses which totalled 2.9% of the entire data collected. For each item, the proportion of neonatal cases for whom guidelines were adhered to, was estimated. The mean and median adherence to protocols per morbidity type were calculated. Total adherence to specified neonatal morbidity protocol was estimated as the sum of scores per theme, presented as a percentage and rated (i.e. adherence status) high, moderate or low if total adherence was 90–100%, 89–60 and < 60% respectively [30]. The difference in total adherence to neonatal protocols within and between the study arms during the time frame of interest was assessed using Wilcoxon rank-sum and permutation tests to determine the significance of these differences due to the small sample size. All analysis were done separately for the two time frames of interest (i.e., 9 months pre and 9 months to the end of the intervention) using two-tailed tests at α = 0.05 in Stata 13 [31].

We analysed the number, and described other activities aimed at improvement in neonatal health outcomes that were undertaken in both the intervention and control clusters during the intervention period.

## Results

One district hospital in each of the four clusters participated in this study. Three of the hospitals were public owned and one was operated by a religious body. There were 2290 deliveries in the intervention arm hospitals and 4440 deliveries in the control arm hospitals in the pre-intervention period. During the intervention period, the number of deliveries stayed about the same in the intervention arm, whereas the number of deliveries increased by 20% in the control arm (Table 1). The number of deliveries per midwife was 76 and 109 in the intervention and control arm respectively during the pre-intervention period. During the intervention period, the number of deliveries per midwife was 66 and 115 in the intervention and control arm respectively. Cluster C recorded the highest delivery related workload during the pre and intervention periods. Table 1 details the characteristics, human resource availability and delivery related workload of the study clusters.

**Table 1:** Distribution of health personnel and delivery related workload in study clusters

**Period**	**Cluster**	**Resource ranking**	**Operating authority**	**Number of doctors**	**Number of obstetricians**	**Number of paediatricians**	**Number of midwives**	**Number of deliveries**
				(n)	(n)	(n)	(n)	
**Pre-trial**	**Intervention arm**							
	A	High -resource	Government	4	0	0	21	1636
	B	Low- resource	Religious	1	0	0	9	654
	**Control arm**							
	C	High -resource	Government	7	0	0	29	3569
	D	Low- resource	Government	2	0	0	10	671
**Trial**	**Intervention arm**							
	A	High -resource	Government	5	0	0	23	1759
	B	Low- resource	Religious	2	1	0	15	735
	**Control arm**							
	C	High -resource	Government	5	0	0	34	4657
	D	Low- resource	Government	3	0	0	13	768
**Period**	**Cluster**	**Resource ranking**	**Deliveries per midwife**	**Deliveries per doctor**	^a^**Workload**	**Proportion of pre and post intervention deliveries**		
**Pre-trial**	**Intervention arm**							
	A	High -resource	78	409	Moderate	0.25		
	B	Low- resource	73	654	Moderate	0.22		
	**Control arm**							
	C	High -resource	123	510	High	0.55		
	D	Low- resource	67	336	Moderate	0.10		
**Trial**	**Intervention arm**							
	A	High -resource	76	352	High	0.22		
	B	Low- resource	49	368	low	0.09		
	**Control arm**							
	C	High -resource	137	931	High	0.59		
	D	Low- resource	59	256	Moderate	0.10		

^a^Estimated by the number of deliveries per midwife; Low < 50, Moderate 50–90, High > 90. The workload in each cluster is higher than the internationally recognized value of 29.5 per midwife. The categorization of workload used here is based solely on comparison between the estimated workload among the study clusters

### Adherence to asphyxia protocols

The prevalence of asphyxia was 3.5 and 15.1 per 1000 deliveries in the intervention and control arm respectively during the pre-intervention period. The 10th and 90th percentile for adherence per theme varied from 0% to 100% in the intervention arm and 33.3% to 100%, in the control arm in this time frame (see Table 2). In the intervention arm, the mean score for total adherence to asphyxia protocols was 35.2% (SD = 5.8%) and in the control arm, it was 52.9% (SD = 16.4%).

**Table 2:** Total adherence score and proportion of asphyxia protocol items adhered to before and during intervention implementation

Protocol item	^a^Pre-trial period (***N*** = 75)		^b^Trial period (***N*** = 66)	
	Intervention n (%)	Control n (%)	Intervention n (%)	Control n (%)
**Diagnosis**				
Diagnosis documented	8 (100.0)	66 (98.5)	14 (93.3)	51 (100.0)
**Signs and symptoms**				
Description of difficulty in breathing	0 (0.0)	14 (20.9)	7 (46.7)	16 (32.0)
Heart rate neonate recorded	0 (0.0)	5 (7.5)	4 (26.7)	48 (94.1)
Tachycardia	0 (0.0)	11 (16.4)	4 (26.7)	48 (94.1)
Respiratory rate	0 (0.0)	45 (67.2)	5 (33.3)	48 (94.1)
Colour of baby	0 (0.0)	25 (37.3)	5 (33.3)	45 (88.2)
APGAR scores written	8 (100.0)	63 (94.0)	14 (93.3)	48 (94.1)
Liquor assessed for meconium staining	2 (25.0)	21 (31.3)	4 (26.7)	8 (15.7)
**Treatment**				
Airway of neonate cleared through suction	1 (12.5)	42 (62.7)	4 (26.7)	14 (27.5)
Warmth provided (using incubator or wrapping)	7 (87.5)	43 (64.2)	2 (13.3)	43 (84.3)
Oxygen given / Bag and mask resuscitation	5 (62.5)	55 (82.1)	9 (60.0)	49 (96.1)
**Total adherence score**				
1	–	–	1 (6.7)	–
2	–	3 (4.5)	3 (20.0)	1 (2.0)
3	2 (25.0)	6 (9.0)	3 (20.0)	2 (3.9)
4	5 (62.5)	7 (10.5)	1 (6.7)	–
5	1 (12.5)	10 (14.9)	3 (20.0)	–
6	–	14 (20.9)	–	–
7	–	13 (19.4)	–	3 (5.9)
8	–	13 (19.4)	–	23 (45.1)
9	–	1 (1.5)	3 (20.0)	15 (29.4)
10	–	–	1 (6.7)	7 (13.73)

^a^There were 2290 and 4440 deliveries in the intervention and control arm respectively in the pre-trial period

^b^There were 2494 and 5425 deliveries in the intervention and control arm respectively in the trial period

During the intervention period, the prevalence of asphyxia was 6 and 9.4 per 1000 deliveries in the intervention and control clusters respectively. The range of values for the 10th and 90th percentile for adherence per theme remained the same during the intervention period. The mean total adherence was 43.6% (SD = 27.5%) in the intervention arm, and in the control arm, it was 74.5% (SD = 14.7%).

**Fig. 1:**
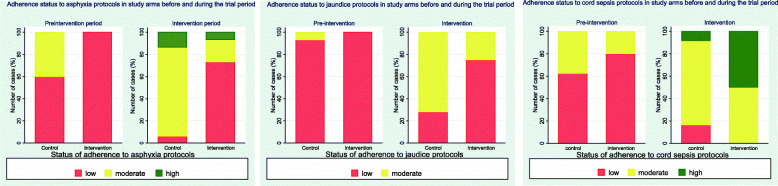
Adherence status per morbidity type before and during the intervention period

**Fig. 2:**
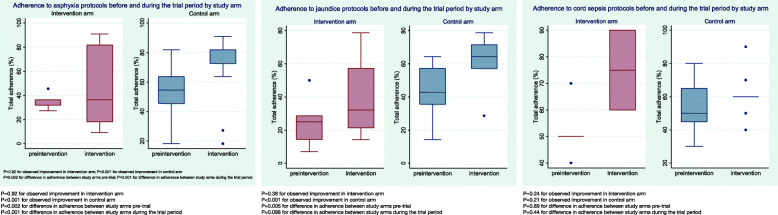
Distribution and change in total adherence to asphyxia, jaundice and cord sepsis protocols among study clusters

Adherence status to asphyxia protocols was moderate to low in the pre-intervention period and high to low in the intervention period (Fig. 1). Overall, there was improvement in total adherence to asphyxia protocols in both study arms (23.9% and 40.8% in the intervention and control clusters respectively). However, improvement in the intervention arm (Fig. 2) was not significant (*p* = 0.92) while improvement in the control arm was significant (*p* < 0.001). Between the study arms, the control arm sites were more adherent to asphyxia protocols compared to the intervention arm before and during the trial period (*p* = 0.002 and *p* < 0.001 respectively).

### Adherence to jaundice protocols

There were 2.6 and 6.5 cases of jaundice per 1000 deliveries in the intervention and control arms respectively during the pre-intervention period. Jaundiced neonates were on average 7.2 days old (SD = 7.3 days). The 10th and 90th percentile for adherence per theme for jaundice protocol varied from 0% to 100% in both study arms (Table 3). The mean total adherence to jaundice protocols was 25.0% (SD = 14.8%) in the intervention arm, and in the control arm it was 45.1% (SD = 12.8%). The cause of jaundice was not identified in 65.7% of cases; in 30.4% of cases, glucose-6-phosphate-dehydrogenase deficiency (G6PD deficiency) was the cause of jaundice and gastroenteritis in one case. Four jaundiced neonates (two in each arm) received no counselling for sunbathing, neither were they put in a phototherapy unit. Two of four neonates in the control arm whose caretakers were advised to give their babies sunbath were not followed up as per protocol. Most jaundiced neonates (33 (91.4%)) received antibiotic as part of their treatment although there was only one documented case of infection (gastroenteritis).

**Table 3:** Total adherence score and proportion of jaundice protocol items adhered to before and during intervention implementation

Protocol item	^a^Pre-trial period (***N*** = 35)		^b^Trial period (***N*** = 54)	
	Intervention n (%)	Control n (%)	Intervention n (%)	Control n (%)
**Diagnosis**				
Diagnosis documented	6 (100.0)	29 (100.0)	4 (100.0)	50 (100.0)
**Signs and symptoms**				
Duration of jaundice stated	0 (0.0)	8 (27.6)	3 (75.0)	46 (92.0)
Temperature checked	3 (50.0)	14 (14.3)	2 (50.0)	11 (22.0)
Assessed for vomiting	1 (25.0)	5 (17.2)	2 (50.0)	1 (2.0)
Assessed for episode(s) of convulsion	1 (25.0)	0 (0.0)	2 (50.0)	0 (0.0)
Assessed for poor feeding	3 (60.0)	3 (10.3)	2 (50.0)	21 (42.0)
Assessed for excessive crying	1 (25.0)	2 (6.9)	1 (25.0)	28 (56.0)
Assessed for hypotonia	0 (0.0)	0 (0.0)	0 (0.0)	2 (4.0)
**Investigation**				
Full blood count done	2 (33.3)	26 (89.7)	2 (50.0)	49 (98.0)
Blood grouping checked	0 (0.0)	22 (75.9)	1 (25.0)	49 (98.0)
Serum bilirubin checked	0 (0.0)	21 (72.4)	1 (25.0)	49 (98.0)
Samples for blood cultures taken	0 (0.0)	8 (27.6)	1 (25.0)	47 (94.0)
Samples for G6PD deficiency screen taken	0 (0.0)	18 (62.1)	0 (0.0)	49 (98.0)
**Treatment**				
Phototherapy given or sunbath advised	4 (66.7)	27 (93.1)	1 (25.0)	50 (100.0)
**Total adherence score**				
1	1 (16.7)	–	–	–
2	1 (16.7)	1 (3.5)	1 (25.0)	–
3	1 (16.7)	2 (6.9)	–	–
4	2 (33.3)	1 (3.5)	1 (25.0)	1 (2.0)
5		4 (13.8)	1 (25.0)	–
6		7 (24.1)	–	–
7	1 (16.7)	5 (17.2)	–	–
8		7 (24.1)	–	13 (26.0)
9		2 (6.9)	–	19 (38.0)
10		–	–	14 (28.0)
11		–	1 (25.0)	3 (6.0)

^a^There were 2290 and 4440 deliveries in the intervention and control arm respectively in the pre-trial period

^b^There were 2494 and 5425 deliveries in the intervention and control arm respectively in the trial period

The number of jaundiced neonates decreased to 1.6 per 1000 deliveries in the intervention arm and increased to 9.2 per 1000 deliveries in the control arm during the intervention period. These jaundiced neonates were on average 5.1 days (SD = 4.5 days) old at the time of the diagnosis. The 10th and 90th percentile for adherence per theme for jaundice protocol varied from 0% to 100% in intervention arm and 14.3% to 100% in the control arm. In the intervention arm, the mean score for total adherence to jaundice protocols was 39.3% (SD = 27.7%); in the control arm it was 64.6% (SD = 8.2%). In 45 (83.3%) of all cases of jaundice, the cause of the jaundice was classified ‘unknown’. One case of physiological jaundice and one case of pathological jaundice were identified in the intervention arm, while one case of cord sepsis and one case of physiological jaundice were identified in the control arm; for the rest of the cases, the cause of the jaundice was not stated in any of the records found in the health facilities. There was no documentation of treatment (using phototherapy or by sun-bathing) of three neonates in the intervention clusters. In the control clusters, all jaundiced neonates received either of the aforementioned treatment options. All sunbathed neonates were followed up during the intervention period. All jaundiced neonates received antibiotics as part of their treatment although only one case of infection (cord sepsis) was identified as a cause of jaundice.

Overall, adherence status was low to moderate during the pre-intervention and intervention periods (Fig. 1). The 57.2% improvement in adherence to jaundice protocols observed in the intervention clusters (Fig. 2) was not significant (*p*-value = 0.38) while improvement observed in the control clusters (43.2%) was significant (*p*-value < 0.001). Comparing the two study arms, control clusters scored higher in adherence to jaundice protocols before and during the intervention period (*p*-value = 0.005 and 0.088 respectively).

### Adherence to cord sepsis protocols

The prevalence of cord sepsis was 2.2 and 1.8 per 1000 deliveries in the intervention and control arms in the pre-intervention period. The average age of these neonates was 5.4 days (SD = 3.8 days). The 10th and 90th percentile for adherence per theme for cord sepsis protocol varied from 16.7% to 100% in both study arms (Table 4). Altogether, the mean total adherence to cord sepsis protocols in the intervention arm was 52.0% (SD = 11.0%) and, 53.8% (SD = 16.0%) in the control clusters.

**Table 4:** Total adherence score and proportion of cord sepsis protocol items adhered to before and during intervention implementation

Protocol item	^a^Pre-trial period (***N*** = 13)		^b^Trial period (***N*** = 14)	
	Intervention n (%)	Control n (%)	Intervention n (%)	Control n (%)
**Diagnosis**				
Diagnosis documented	5 (100.0)	8 (100.0)	2 (100.0)	12 (100.0)
**Signs and symptoms**				
Cord assessed for odor, pus and wetness	3 (60.00)	2 (25.0)	1 (50.0)	9 (75.0)
Skin around cord assessed for redness	2 (40.0)	3 (37.5)	0 (0.0)	4 (33.3)
Assessment for fever	3 (60.0)	5 (62.5)	1 (50.0)	3 (25.0)
Heart rate, pulse rate, respiratory rate	2 (40.0)	7 (87.5)	2 (100.0)	11 (91.7)
Abdomen palpated	0 (0.00)	2 (25.0)	2 (100.0)	5 (41.7)
Conjunctiva or haemoglobin checked	0 (0.00)	1 (12.5)	2 (100.0)	1 (8.3)
**Treatment**				
Cord hygiene education given to mother	2 (40.0)	0 (0.0)	1 (50.0)	5 (41.7)
Antibiotics given	5 (100.0)	8 (100.0)	2 (100.0)	12 (100.0)
Monitoring of vitals	4 (80.0)	7 (87.5)	2 (100.0)	11 (91.7)
**Total adherence score**				
1	–	–	–	–
2	–	–	–	–
3	–	1 (12.5)	–	–
4	1 (20.0)	1 (12.5)	–	1 (8.3)
5	3 (60.0)	3 (37.5)	–	1 (8.3)
6		1 (12.5)	1 (50.0)	8 (66.7)
7	1 (20.0)	1 (12.5)	–	1 (8.3)
8		1 (12.5)	–	–
9		–	1 (50.0)	–
10		–	–	1 (8.3)

^a^There were 2290 and 4440 deliveries in the intervention and control arm respectively in the pre-trial period

^b^There were 2494 and 5425 deliveries in the intervention and control arm respectively in the trial period

There were 0.8 and 2.2 cases of cord sepsis per 1000 deliveries during intervention implementation. The average age of these neonates was 5.9 days (SD = 4.9 days). Altogether, the mean total adherence to cord sepsis protocols in the intervention arm was 75.0% (SD = 21.2%) whereas, in the control arm, it was 60.8% (SD = 11.7%). The 10th and 90th percentile for adherence per theme for cord sepsis protocol varied from 50% to 100% in the intervention arm and 33.3% to 100% in the control arm.

Adherence status was low to moderate during the pre-intervention and intervention periods (Fig. 1). Improvement in adherence to protocols was observed in both study arms (44.2% and 13% in intervention and control arms respectively) (Fig. 2). However, these improvements were not significant (*p* = 0.24 and *p* = 0.21 for intervention and control arms respectively). Between the study arms, adherence to cord sepsis protocols were not significantly different (*p* = 0.89 and *p* = 0.44 for intervention and control arms respectively).

### Concurrent neonatal health activities in clusters

In the intervention clusters, there were two training programmes that were aimed at improving neonatal health outcomes during the intervention period while in the control clusters, training programmes aimed at improving neonatal health numbered more than 12 (Table 5). Eight of these trainings (two in the intervention clusters and six in the control clusters) were intensive exercises aimed at improving new-born resuscitation and lasted six (6) to seven (7) days. These intensive training programmes were organized by a non-governmental agency. The rest of the training programmes in the control clusters usually lasted for one (1) day.

**Table 5:** Concurrent neonatal health improvement activities in study clusters during the intervention period

Arm	Cluster	Resource ranking	Total number of activities	Activities/topics discussed
**Intervention**	A	High	1	Making every baby count initiative
**Intervention**	B	Low	1	Making every baby count initiative
**Control**	C	High	> 7	Policy on breast feeding and Hepatitis exposed babies; Assisted Vacuum Delivery; ^a^Helping babies breathe training; Bi-weekly continuous professional training aimed at reducing the incidence of birth asphyxia and improving new-born resuscitation
**Control**	D	Low	5	Accelerating the achievement of Millennium Development Goal 4; Provider training; Helping babies breathe and essential care for every baby; 7th District Hospital provider training; Maternal and Neonatal audit workshop

^a^ There were a total of at least 5 rounds of this training with a new group of midwives being trained each time

## Discussion

### Adherence to asphyxia protocols

We observed fairly good adherence to asphyxia diagnosis protocol in this study. However, adherence to ‘signs and symptoms’ protocols was sub-optimal in both study clusters particularly in the pre-intervention period. Several of the ‘signs and symptoms’ assessments culminates in the APGAR score of neonates [32]. Not assessing these signs and symptoms can lead to inaccurate APGAR scores and inappropriate treatment of neonates who require resuscitation. Surprisingly, the APGAR scores were usually documented, thus one could argue that these signs and symptoms assessments were done but not documented because the natural focus is to treat the patient and not record [33], however, video recording of neonatal resuscitation has shown otherwise [33–35]. In the intervention clusters, adherence to treatment protocols worsened during the intervention period. Lack of knowledge about asphyxia as documented in Malawi could be an explanation for this observation [36]. The intervention (mCDMSS) was intended to bridge such knowledge gap, however, the absence of knowledge transfer (about the intervention) implies persistence of lack of knowledge and access gap in the intervention clusters possibly through suboptimal use of the intervention [37]. Monthly reminders concerning the availability of the mCDMSS for the use of health workers and, re-training of health workers at post in health facilities during supervisory visits by the project team, seem not to have been effective in addressing the challenge of suboptimal use of the intervention [37]. Poor adherence as observed suggests focused support for health workers in the management of asphyxia in order to improve adherence to its protocols.

### Adherence to jaundice protocols

Protocols for management of Jaundice were least adhered to among the three morbidity protocols understudied. There was poor adherence to protocol items for the theme ‘Signs and symptoms’ of jaundice in all clusters in both time frames. Of note is the assessment of neonates for convulsion and hypotonia. While the diagnosis of convulsion may be difficult in neonates [38, 39], hypotonia can be objectively assessed; the lack of documented evidence of assessment of these two critical signs of the central nervous system (CNS) is undesirable given disabilities associated with CNS complications (kernicterus) from jaundice [40]. Failure of the health workers to recognize at-risk infants and poor management of hyperbilirubinemia is a known cause of kernicterus [40]. The observed complete non-adherence to jaundice investigation protocols in the intervention arm during the pre-intervention period and poor adherence to these protocols in the intervention period could be due to the absence of the rapid tests or laboratory equipment to run these tests in the hospitals. Lack of required equipment is associated with non-adherence to protocols [41–43]. Neonates with mild jaundice may have been the ones not treated in this study; the absence of follow-up of jaundiced neonates has been previously documented and can be associated with dire consequences should the jaundice worsen [40]. We report indiscriminate use of antibiotics in cases of jaundice and this suggests the need for training on rational use of antibiotics in the study setting.

### Adherence to cord sepsis protocols

Prevalence of cord sepsis in this study was low. This may be due to on-going interventions in the GHS to promote good cord hygiene practises [44, 45]. This low prevalence of cord sepsis may however lead to poor recall of assessments for cord sepsis cases as observed in the low adherence score for ‘signs and symptoms’ in this study. Local signs of cord sepsis (e.g. pus and odour) are associated with mortality [46], therefore lack of assessment for these local ‘signs and symptoms’ can be potentially catastrophic for neonates as important complications from the cord infection that may warrant urgent attention or treatment modification may be missed. All cases of cord sepsis were treated with antibiotics which indicates that once a diagnosis is made, treatment will be initiated, and the vitals of patients will be monitored as observed. The non-adherence to protocol item regarding cord hygiene education for caregivers presents a missed opportunity to teach cord hygiene in a setting where poor cord hygiene still exists in some communities [47]. Caregivers are known to be inappropriately educated by health workers about the morbidities, treatment and associated complications their wards may experience [40, 41].

### Adherence to protocols in general

We found no case of complete adherence to protocols for all three morbidity types (asphyxia, jaundice, and cord sepsis) in this study using data of 257 neonates in the four district hospitals. A similar observation was made in another study where every resuscitation had an error [33]. Improvement in adherence to all three morbidity type protocols during the intervention period in both the control and intervention arms is possibly due to training programmes of the GHS and her partners in this area. Such efforts must be documented and reviewed to optimize their effect on improvement in neonatal healthcare services. Cluster C recorded the highest proportion of deliveries, and the highest workload but the best adherence to protocols before and during the intervention period. Low workloads can influence competence and high workloads can influence ability to respond adequately; the high workload of cluster C could have positively influenced the cluster’s observed adherence to protocols.

### Contribution of concurrent activities to improvements adherence to neonatal care protocols

“Even if you know everything you can forget” [48]. Frequent reminders, trainings and refresher trainings are a means to improve health outcomes in general. The observed higher improvements in adherence to protocols in the control clusters compared to the intervention clusters may reflect differences in knowledge across the intervention and control clusters resulting from the training programmes that were more frequently undertaken by hospital management, the GHS and her partners in control clusters. Commensurate efforts on neonatal health improvement training programmes in addition to the mCDMSS in the intervention clusters may have led to significant improvements in adherence to protocols in the intervention clusters as well. The higher number of concurrent neonatal health improvement activities in the control clusters could also explain the observed lower odds of neonatal death in the main CRCT findings [49]**.**

### Limitation

We sought to understand the pattern of health worker adherence to neonatal health protocols before and during the implementation of an mCDMSS, but our study has certain limitations. Differences in adherence to protocols by resource allocation type per study arm were not assessed. Due to the low incidence of cases among the various subgroups and because individual level data concerning mortality was incomplete in the DHIMS2 and in the health facility medical records, we could not control for the effect of the concurrent neonatal improvement activities in our analysis, nor analyse the relationship between adherence and neonatal mortality as originally indicated in our trial registration. The results of our study should therefore be interpreted in the light of these methodological limitations**.** We did not evaluate the type of health care provider in relation to the care provided nor the factors that may have influenced adherence to neonatal protocols. Qualitative analyses of why the observed pattern of adherence occurred could have provided more insight into the results we have obtained and are recommended for future studies.

## Conclusion

The question of whether mHealth interventions can improve adherence to neonatal health protocols in a low-resource setting remains difficult to answer from the evidence generated in this study, but, during the study, adherence improved irrespective of intervention allocation. This was particularly observed for the control clusters, and concurrent neonatal improvement interventions that took place in the study clusters may explain this effect. It is therefore essential to document and review all ongoing interventions whose goals are to improve health worker adherence to neonatal health protocols in study settings. Concurrent neonatal health improvement activities must be taken into account in similar mHealth evaluations. Future studies should relate adherence with patient outcomes.

## Supplementary information

**Additional file 1.**

## Data Availability

Data for this study is available on request by emailing the corresponding author at ansomaame@hotmail.com.

## Abbreviations

CDMS: Clinical decision-making support
CHPS: Community based health planning and services
CNS: Central nervous system
CRCT: Cluster randomized controlled trial
CUG: Closed user group
DHIMS2: District health information management system 2
G6PD: Glucose-6- phosphate dehydrogenase deficiency
GHS: Ghana health service
HCs: Health centres
mCDMSS: Mhealth clinical decision-making support system
SIM: Subscriber identification module
SMS: Short message system
USSD: Unstructured supplementary service data
