# Supplementary material for: Can an mhealth clinical decision-making support system improve adherence to neonatal healthcare protocols in a low-resource setting?
Source: BMC Pediatr. 2020 Nov 27;20:534. doi: 10.1186/s12887-020-02378-1 (PMC7694934; doi:10.1186/s12887-020-02378-1)
Supplement: Supplementary file 1 — Additional file 1. [file 12887_2020_2378_MOESM1_ESM.docx]

# **Appendix I: Type of medical records from which data was extracted before and during intervention implementation**

| **^*^Type of medical record** | **Morbidity type** | | |
| --- | --- | --- | --- |
|  | **Asphyxia** | **Jaundice** | **Cord sepsis** |
|  | **n (%)** | **n (%)** | **n (%)** |
|  |  |  |  |
| Admissions and discharge book | 99 (70.2) | 63 (70.8) | 20 (74.5) |
| Asphyxia book | 8 (5.7) | - | - |
| Clinical notes | 2 (1.4) | 2 (2.3) | - |
| Delivery book | 41 (28.1) | 1 (1.1) | 1 (3.7) |
| Lab results | - | 1 (1.1) | - |
| Neonatal intensive care unit form | 5 (3.6) | - | - |
| New-born examination form | 1 (0.7) | - | - |
| Nurses notes | 26 (18.4) | 55 (61.8) | 12 (44.4) |
| Patient folder | 63 (44.7) | 19 (21.2) | 16 (59.30) |
| Patient information sheet | 14 (9.9) | 10 (11.2) | 6 (22.2) |
| Postnatal book | 1 (0.7) | - | 27 (100.0) |
| Referral book | 5 (3.6) | - | 1 (3.7) |
| Regulation form | 3 (2.1) | - | - |
| Report book | 100 (70.9) | 57 (64.0) | 11 (40.7) |
| Summary or Labour form | 31 (22.0) | 9 (10.1) | 4 (14.8) |

^*^Medical records include both Ghana Health Service recognized registers and registers or books used locally in the hospitals to collect data as deemed relevant by the individual hospital management

# **Appendix IIa: Number of ‘don’t know’ responses to asphyxia protocol items before and during intervention implementation**

| **Protocol item** | **^†^Pre-intervention period** | | **^ⱡ^Intervention period** | |
| --- | --- | --- | --- | --- |
|  | **Intervention**  **n (%)** | **Control**  **n (%)** | **Intervention**  **n (%)** | **Control**  **n (%)** |
| **Diagnosis** |  |  |  |  |
| Diagnosis documented | 0 (0.0) | 0 (0.0) | 0 (0.0) | 0 (0.0) |
| **Signs and symptoms** |  |  |  |  |
| Description of difficulty in breathing | 0 (0.0) | 0 (0.0) | 0 (0.0) | 6 (12.0) |
| Heart rate neonate recorded | 0 (0.0) | 0 (0.0) | 3 (20.0) | 0 (0.0) |
| Tachycardia | 0 (0.0) | 0 (0.0) | 3 (20.0) | 1 (2.0) |
| Respiratory rate | 0 (0.0) | 0 (0.0) | 3 (20.0) | 1 (2.0) |
| Colour of baby | 0 (0.0) | 0 (0.0) | 3 (20.0) | 1 (2.0) |
| APGAR scores written | 0 (0.0) | 0 (0.0) | 0 (0.0) | 0 (0.0) |
| Liquor assessed for meconium staining | 0 (0.0) | 0 (0.0) | 3 (20.0) | 9 (17.7) |
| **Treatment** |  |  |  |  |
| Airway of neonate cleared through suction | 0 (0.0) | 0 (0.0) | 3 (20.0) | 6 (11.8) |
| Warmth provided (using incubator or wrapping) | 0 (0.0) | 0 (0.0) | 2 (20.0) | 3 (3.9) |
| Oxygen given / Bag and mask resuscitation | 0 (0.0) | 0 (0.0) | 3 (20.0) | 1 (2.0) |
| **Total missing data** | **0 (0.0)** | **0 (0.0)** | **23 (13.9)** | **28 (5.0)** |

# **Appendix IIb: Number of ‘don’t know’ responses to jaundice protocol items before and during intervention implementation**

| **Protocol item** | **^†^Pre-intervention period** | | **^ⱡ^Intervention period** | |
| --- | --- | --- | --- | --- |
|  | **Intervention**  **n (%)** | **Control**  **n (%)** | **Intervention**  **n (%)** | **Control**  **n (%)** |
|  |  |  |  |  |
| **Diagnosis** |  |  |  |  |
| Diagnosis documented | 0 (00.0) | 0 (0.00) | 0 (0.0) | 0 (0.00) |
| **Signs and symptoms** |  |  |  |  |
| Duration of jaundice stated | 0 (0.0) | 0 (0.0) | 0 (0.0) | 0 (0.0) |
| Temperature checked | 0 (0.0) | 0 (0.0) | 0 (0.0) | 0 (0.0) |
| Assessed for vomiting | 0 (0.0) | 0 (0.0) | 0 (0.0) | 4 (8.0) |
| Assessed for episode(s) of convulsion | 0 (0.0) | 0 (0.0) | 0 (0.0) | 4 (8.0) |
| Assessed for poor feeding | 0 (0.0) | 0 (0.0) | 0 (0.0) | 4 (8.0) |
| Assessed for excessive crying | 0 (0.0) | 0 (0.0) | 0 (0.0) | 4 (8.0) |
| Assessed for hypotonia | 0 (0.0) | 0 (0.0) | 0 (0.0) | 0 (0.0) |
| **Investigation** |  |  |  |  |
| Full blood count done | 0 (0.0) | 0 (0.0) | 0 (0.0) | 0 (0.0) |
| Blood grouping checked | 0 (0.0) | 0 (0.0) | 0 (0.0) | 0 (0.0) |
| Serum bilirubin checked | 0 (0.0) | 0 (0.0) | 0 (0.0) | 0 (0.0) |
| Samples for blood cultures taken | 0 (0.0) | 0 (0.0) | 0 (0.0) | 0 (0.0) |
| Samples for G-6-P-D deficiency screen taken | 0 (0.0) | 0 (0.0) | 0 (0.0) | 0 (0.0) |
| **Treatment** |  |  |  |  |
| Phototherapy given or sunbath advised | 0 (0.0) | 0 (0.0) | 0 (0.0) | 0 (0.0) |
| **Total missing data** | **0(0.0)** | **0 (0.0)** | **0 (0.0)** | **16 (2.3)** |
|  |  |  |  |  |

# **Appendix IIc: Number of ‘don’t know’ responses to cord sepsis protocol items before and during intervention implementation**

| **Protocol item** | **^†^Pre-intervention period** | | **^ⱡ^Intervention period** | |
| --- | --- | --- | --- | --- |
|  | **Intervention5**  **n (%)** | **Control8**  **n (%)** | **Intervention2**  **n (%)** | **Control12**  **n (%)** |
| **Diagnosis** |  |  |  |  |
| Diagnosis documented | 0 (0.0) | 0 (0.0) | 0 (0.0) | 0 (0.0) |
| **Signs and symptoms** |  |  |  |  |
| Cord assessed for odour, pus and wettness | 0(0.0) | 1 (12.5) | 1 (50.0) | 2 (16.7) |
| Skin around cord assessed for redness | 0 (0.0) | 2 (25.0) | 1 (50.0) | 8 (66.7) |
| Assessment for fever | 0(0.0) | 1 (12.5) | 0 (0.0) | 0 (0.0) |
| Heart rate, pulse rate, respiratory rate | 0(0.0) | 1 (12.5) | 0 (0.0) | 1 (8.3) |
| Abdomen palpated | 0 (0.0) | 0 (0.0) | 0 (0.0) | 0 (0.0) |
| Conjunctiva or haemoglobin checked | 0 (0.00) | 0 (0.0) | 0 (0.0) | 2 (16.7) |
| **Treatment** |  |  |  |  |
| Cord hygiene education given to mother | 0 (0.0) | 0 (0.0) | 0 (0.0) | 0 (0.0) |
| Antibiotics given | 0 (0.0) | 0 (0.0) | 0 (0.0) | 0 (0.0) |
| Monitoring of vitals | 0 (0.0) | 1 (8.3) | 0 (0.0) | 0 (0.0) |
| **Total missing data** | **0 (0.0)** | **6 (7.5)** | **2 (10.0)** | **13 (10.8)** |
|  |  |  |  |  |
